# Maleic Acid – but Not Structurally Related Methylmalonic Acid – Interrupts Energy Metabolism by Impaired Calcium Homeostasis

**DOI:** 10.1371/journal.pone.0128770

**Published:** 2015-06-18

**Authors:** Ali Tunç Tuncel, Thorsten Ruppert, Bei-Tzu Wang, Jürgen Günther Okun, Stefan Kölker, Marina Alexandra Morath, Sven Wolfgang Sauer

**Affiliations:** Department of General Pediatrics, Division of Inborn Metabolic Diseases, University Children’s Hospital, Heidelberg, Germany; Mayo Clinic, UNITED STATES

## Abstract

Maleic acid (MA) has been shown to induce Fanconi syndrome via disturbance of renal energy homeostasis, though the underlying pathomechanism is still under debate. Our study aimed to examine the pathomechanism underlying maleic acid-induced nephrotoxicity. Methylmalonic acid (MMA) is structurally similar to MA and accumulates in patients affected with methymalonic aciduria, a defect in the degradation of branched-chain amino acids, odd-chain fatty acids and cholesterol, which is associated with the development of tubulointerstitial nephritis resulting in chronic renal failure. We therefore used MMA application as a control experiment in our study and stressed hPTECs with MA and MMA to further validate the specificity of our findings. MMA did not show any toxic effects on proximal tubule cells, whereas maleic acid induced concentration-dependent and time-dependent cell death shown by increased lactate dehydrogenase release as well as ethidium homodimer and calcein acetoxymethyl ester staining. The toxic effect of MA was blocked by administration of single amino acids, in particular L-alanine and L-glutamate. MA application further resulted in severe impairment of cellular energy homeostasis on the level of glycolysis, respiratory chain, and citric acid cycle resulting in ATP depletion. As underlying mechanism we could identify disturbance of calcium homeostasis. MA toxicity was critically dependent on calcium levels in culture medium and blocked by the extra- and intracellular calcium chelators EGTA and BAPTA-AM respectively. Moreover, MA-induced cell death was associated with activation of calcium-dependent calpain proteases. In summary, our study shows a comprehensive pathomechanistic concept for MA-induced dysfunction and damage of human proximal tubule cells.

## Introduction

Renal proximal tubule epithelial cells play a central role in filtration, secretion, and resorption of diverse ions and metabolites [1]. These processes require high amounts of molecular energy, provided by numerous mitochondria located on the basolateral side of the proximal tubule. Accumulation of nephrotoxic substances as well as an imbalanced energy production disturb tubular transport and result in renal damage. Patients with inherited deficiency of energy metabolism often present with De Toni-Debré-Fanconi syndrome or–to a lesser degree–tubulointerstitial nephritis. Primary mitochondriopathies, such as respiratory chain defects, are often complicated by De Toni-Debré-Fanconi syndrome [2, 3] in which ATP depletion is likely to damage proximal tubule. However, some patients develop tubulointerstitial nephritis [4–6]. It remains to be unravelled which additional factors precipitate-Fanconi syndrome or tubulointerstitial nephritis.

Noteworthy, treatment of proximal tubules *in vitro* and *in vivo* with maleic acid (MA) is used as a model system to investigate Fanconi syndrome. MA has been indicated to (1) directly and indirectly inhibit Na^+^-K^+^-ATPases [7, 8], (2) disturb overall mitochondrial function [9–11], (3) fatty acid metabolism [12], (4) disturb membrane transport processes of the endoplasmic reticulum [13], (5) and its toxic effect was linked to calcium [12]. Though MA treatment is a well-established model to study Fanconi syndrome, the underlying pathomechanism is still under debate. The main aim of our study was therefore to examine the nephrotoxic effects of MA on hPTECs *in vitro* to induce Fanconi syndrome and to examine the underlying mechanism. The structurally related methylmalonic acid (MMA) accumulates in methylmalonic acidurias, an etiologically heterogeneous group of inherited metabolic diseases caused by defects of the mitochondrial enzyme methylmalonyl-CoA mutase and/or mutations of vitamin B_12_ metabolism and transport. The defect is localized in the degradation of branched-chain amino acids, odd-chain fatty acids and cholesterol. Untreated patients with this disease are often affected by metabolic crisis and death due to multi-organ failure. Patients who survive these life-threatening crises or those with a milder disease course are prone to develop chronic renal failure due to tubulointerstitial nephritis [14–16] but not Fanconi syndrome. MA (*cis*-butenedioic acid) and MMA (2-methylpropanedioic acid) share structural similarities with the citric acid cycle intermediates succinic acid (butanedioic acid) and fumaric acid (butenedioic acid). It has therefore been speculated that accumulation of MA and MMA may interfere with energy metabolism. However, both dicarboxylic acids are associated with distinct renal pathologies (Fanconi syndrome vs. tubulointerstitial nephritis) suggesting unequal underlying pathomechanisms. We therefore conducted treatment with MMA as a control experiment to further underline the specificity of our findings.

## Material and Methods

### Cell culture

Human proximal tubule epithelial cells (hPTECs) were purchased from Clonetics (Lonza, Basel, Switzerland) and cultivated in DMEM/Ham’s F-12 (purchased from PAA, Pasching, Austria) supplemented with 10% heat-inactivated FCS, 100 U/ml penicillin, 0.1 mg/ml streptomycin, 5 µg/ml insulin, 5 µg/ ml transferrin, 35 ng/ml hydrocortisone, 5 ng/ml hEGF, 6.4 mg/ml T_3_, 5 ng/ml selenite and 3.4 mg/ml β-NAD. The cells were incubated in an incubator for 2 days at 37°C and 5% CO_2_ and the medium was renewed after 24 hours. The cells were subcultivated after reaching a confluency of approximately 70–80%.

### Protein analysis

Total protein was determined using Bio-Rad DC Protein Assay. Results of all experiments were normalized to total protein and given as nmol/mg.

### Treatment protocol

Human proximal tubule epithelial cells (hPTECs) were seeded into 24-well-plates (4x10^4^/well) and cultivated for 2 days to confluency in DMEM. The medium was then discarded and 1 ml of pre-warmed Krebs-Ringer-Buffer (KRB) containing the corresponding chemical was added to the cells. Following this procedure the 24-well-plates were placed into an incubator (37°C) and cells were treated for 24 hours. For biochemical and bioenergetical studies, cells were washed twice after treatment to remove dead cells and only the remaining cell were subject to further analysis.

### Lactate dehydrogenase (LDH) release

After 24 hours of treatment LDH activity in the supernatants was measured in a buffer consisting of 0.2 M Tris/HCl, 6.6 mM β-NADH and 30 mM sodium pyruvate (pH 7.3).

### Preparation of sub-cellular fractions

Cells were disrupted using a 27x ½” needle in ice-cold buffer A consisting of 250 mM sucrose, 50 mM KCl, 5 mM MgCl_2_, and 20 mM Tris (pH 7.4) and the homogenates were centrifuged at 600×*g*, 4°C for 10 min. For preparation of “mitochondria-enriched” fractions, the 600×*g*_*max*_ supernatant was centrifuged 10 min, 4°C at 3,500×*g* (pellet). For preparation of cytosolic (supernatant) fraction the 600×*g*_*max*_ supernatant was centrifuged at 11,000×*g*, 4°C for 20 min.

### Single enzyme assays of glycolysis, citric acid cycle and respiratory chain

Steady-state activities were determined using a computer-tunable spectrophotometer (Spectramax Plus Microplate Reader, Molecular Devices, Sunnyvale, California, United States) operating in the dual wavelength mode; samples were analyzed in temperature-controlled 96-well plates in a final volume of 300 μl. Glycolytic enzymes, citric acid cycle enzymes and respiratory chain complexes were analyzed as previously described [17–21].

### Oxygen consumption

Mitochondrial respiratory rate was measured according to a previously described protocol [22] using computer-supported high-resolution oxygen electrode (Oroboros 1 oxygraph system, Paar, Innsbruck, Austria).

### Succinate and pyruvate respiration

^14^CO_2_ production of cells respiring on [1-^14^C] pyruvate and [1,4-^14^C] succinate were determined as previously described [17].

### Quantitative analysis of acylcarnitines and amino acids

Acylcarnitines and amino acids were determined in cell homogenates by electrospray ionization tandem mass spectrometry (MS/MS) according to a modified method described by Sauer et al. [23].

### CAM & EHD staining

Simultaneous staining with calcein acetoxymethyl ester (CAM) and ethidium homodimer (EHD) developed by Haugland et al. (1994) (US Patent Nr. 5,314,805) was used to distinguish between viable and non-viable cells after treatment. Concentrations of 0.5 µM and 2 µM of calcein-AM and ethidium homodimer respectively were used to stain the treated cells. HPTECs were incubated for 15 minutes with both dyes.

### Annexin V / PI staining and FACS

Human proximal tubule epithelial cells (hPTECs) were processed using an Annexin V apoptosis assay kit (BD Annexin V Apoptosis Detection Kit) according to the manufacturer’s protocol (BD Biosciences) and have been analyzed using flow cytometry.

### Statistical analysis

Control and experimental groups were compared by unpaired Student's *t*-test or by repeated measures ANOVA (rANOVA) for concentration dependent experiments. All statistical analyses were performed by SPSS for Windows 16.0 Software. The corresponding *p*-values have been mentioned below the supplementary tables for each figure.

## Results

### Maleic acid but not methylmalonic acid induces cell death in hPTEC

**Fig 1:**
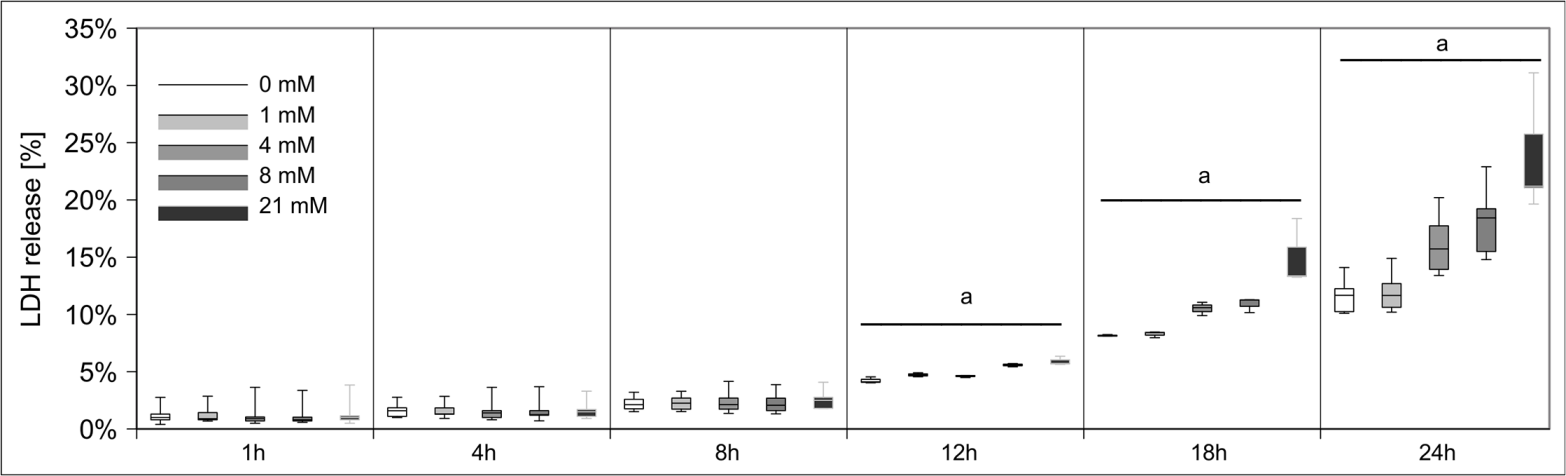
Induction and prevention of hPTEC damage by MA. hPTECs were stressed with increasing amounts of MA (0, 1, 4, 8, 21 mM) for up to 24h. MA led to a concentration- and time- dependent LDH release. Data are presented as percent of untreated control of n = 20 independent experiments.

**Fig 2:**
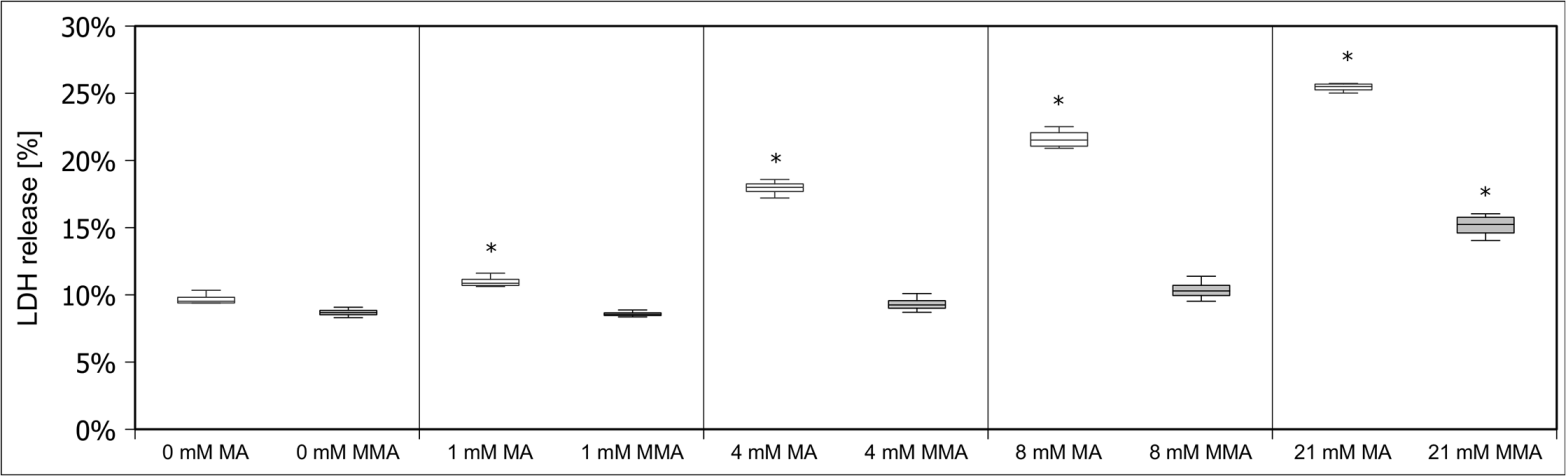
Induction and prevention of hPTEC damage by MMA. hPTECs were stressed with increasing amounts of MMA (0, 1, 4, 8, 21 mM) for up to 24h. In contrast to MA, MMA only influenced cell vitality in higher concentrations. Data are presented as percent of untreated control of n = 4 independent experiments.

Initially, we examined the cytotoxic effects of MA on hPTECs. LDH release was determined as late but stable marker of cell death. To prove the specificity of the effects of MA on these cells, we first performed parallel experiments with structurally similar succinic acid and fumaric acid. Since succinic acid and fumaric acid are metabolized via the citric acid cycle and since their carbon backbones are used for intracellular production of amino acids and other compounds the use of these dicarboxylic acids as negative controls might be disadvantageous. We therefore also tested MMA as a negative control. HPTECs were exposed to increasing MA and MMA concentrations (0, 1, 4, 8, 21 mM ~ 0, 0.1, 0.5, 1, 2.5 mg/ml of MA/MMA) for up to 24 h. These concentrations have been chosen according to previously published protocols. For hPTECs are equipped with a functional methylmalonyl-CoA mutase, we first tested whether the applied MMA was metabolized. Importantly, MMA concentrations in medium did not decrease during the incubation period. MA, on the other hand, induced a concentration and timedependent increase in LDH release **(Fig 1)**. In contrast MMA revealed only a slight toxicity at the highest tested concentration **(Fig 2)**. Moreover, MMA did not affect any of the further investigated biochemical or bioenergetical parameters (data not shown).

**Fig 3:**
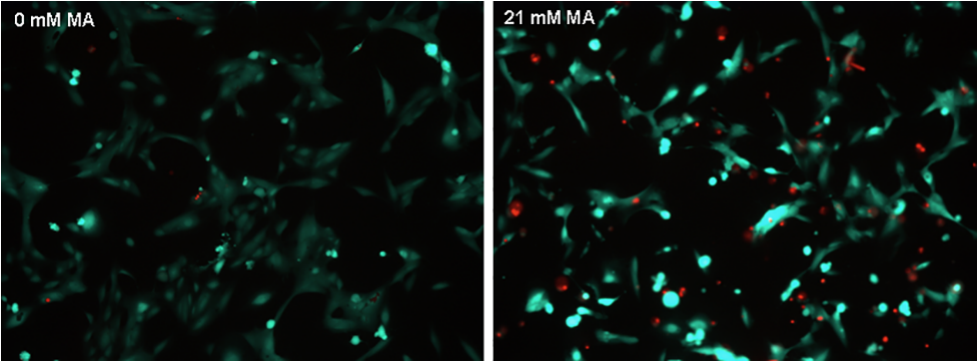
CAM and EHD stainings before and after MA treatment. CAM and EHD stainings revealed that MA treatment decreased hPTEC vitality already after 5h. Data are presented as percent of untreated control of n = 4 independent experiments.

Since LDH is a late marker of cell death, we examined cell viability using stainings with calcein-AM (CAM) and ethidium homodimer (EHD). Already after 5 hours of treatment dying cells could be observed **(Fig 3)**.

### Maleic acid leads to cell death through activation of apoptosis pathways

**Fig 4:**
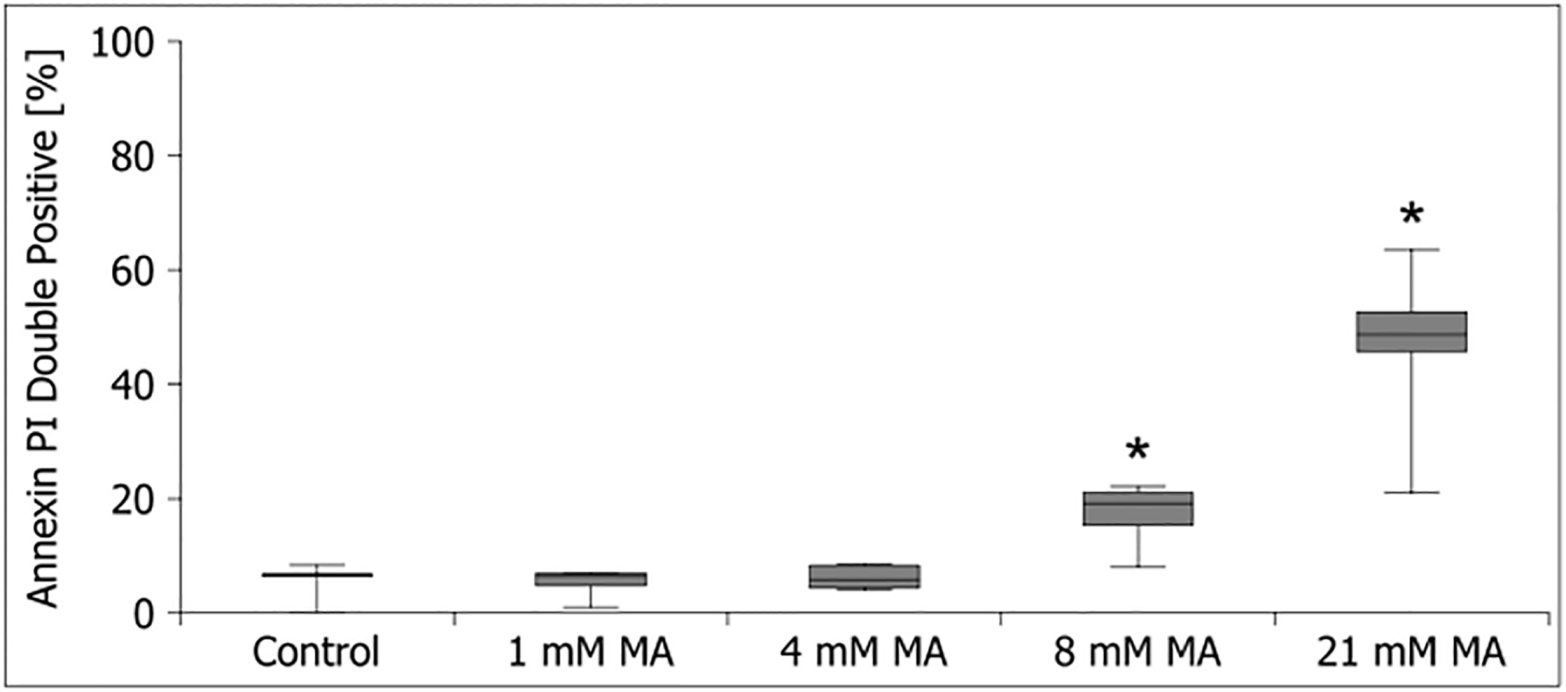
Activation of apoptosis pathways after MA treatment in hPTECs. Increasing concentrations of MA led to more cell death and the activation of apoptosis pathways could be shown with annexin V–PI staining. Data are presented as percent of untreated control of n = 4 independent experiments.

In order to further examine the pathomechanism underlying MA cytotoxicity we used annexin V-FITC and PI staining to differentiate between necrosis and apoptosis. HPTECs have been treated with increasing concentrations of MA and were then stained using annexin V-FITC and PI after 24 hours of treatment. The cells have then been analysed with flow cytometry (FACS). MA treatment led to a concentration dependent activation of apoptosis pathways in higher concentrations **(Fig 4)**.

### Rescue of maleic acid-induced cell death by anaplerotic amino acids and inhibition of organic anion transporter

**Fig 5:**
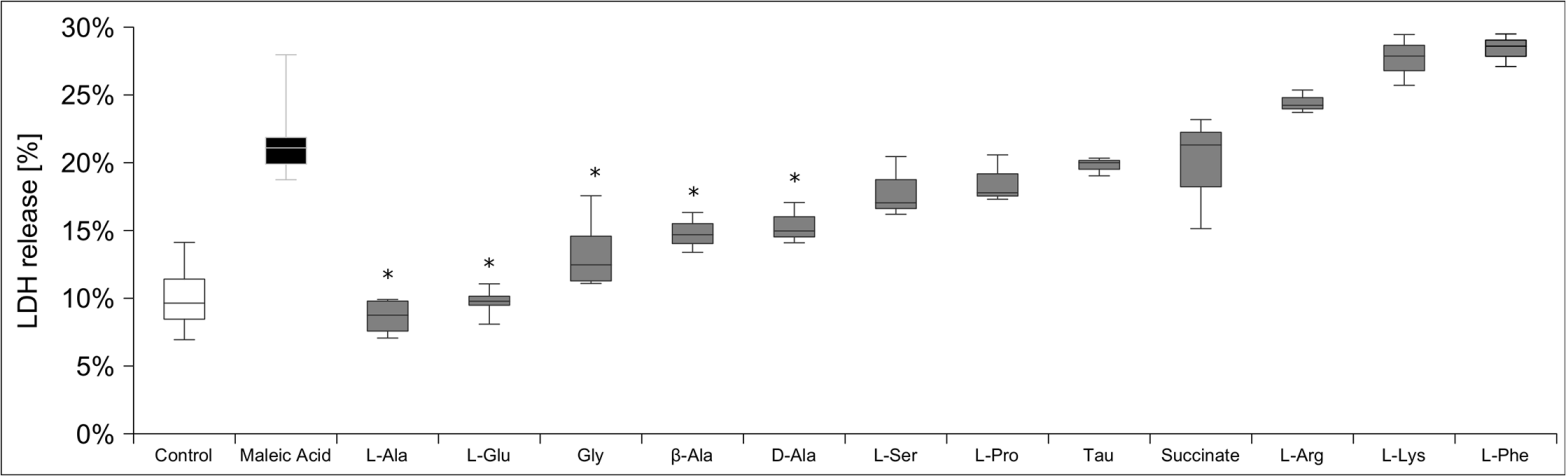
Effects of diverse metabolites on MA cytotoxicity. To prevent MA-toxicity hPTEC were cultivated for 24h with 21 mM MA +/- diverse amino acids (each 5 mM) and substrates for organic anion transporters (each 2mM). L-Alanine (Ala) and L-glutamate (Glu) prevented MA induced LDH release. There was however no significant difference between treatment with L-Alanine (Ala) and L-glutamate (Glu) (*5*). L-Glycine (Gly), D- and β-alanine diminished MA toxicity, whereas L-serine and L-proline (Pro), taurine (Tau), L-arginine (Arg), L-lysine (Lys) and L-phenylalanine (Phe) had no effect. Data are presented as percent of untreated control of n = 4 independent experiments.

It has previously been shown that glycine and structural similar amino acids reduce or even prevent damage to proximal tubule by hypoxia and toxic agents by a not yet elucidated mechanism [24]. Therefore we tested the cytoprotective effect of several amino acids on MA toxicity (21 mM MA ± 5 mM amino acid for 24 h). Strikingly, L-alanine and L-glutamate fully abolished the toxic effect of MA **(Fig 5)** indicating an anaplerotic rescue mechanism. However L-glycine, D-alanine and β-alanine also reduced LDH release, whereas L-serine and L-proline as well as the longer amino acids taurine, L-arginine, L-lysine and L-phenylalanine did not exert any protective effects. Also succinic acid, that has previously been described to act protective [12], did not affect MA-induced LDH release in hPTECs.

**Fig 6:**
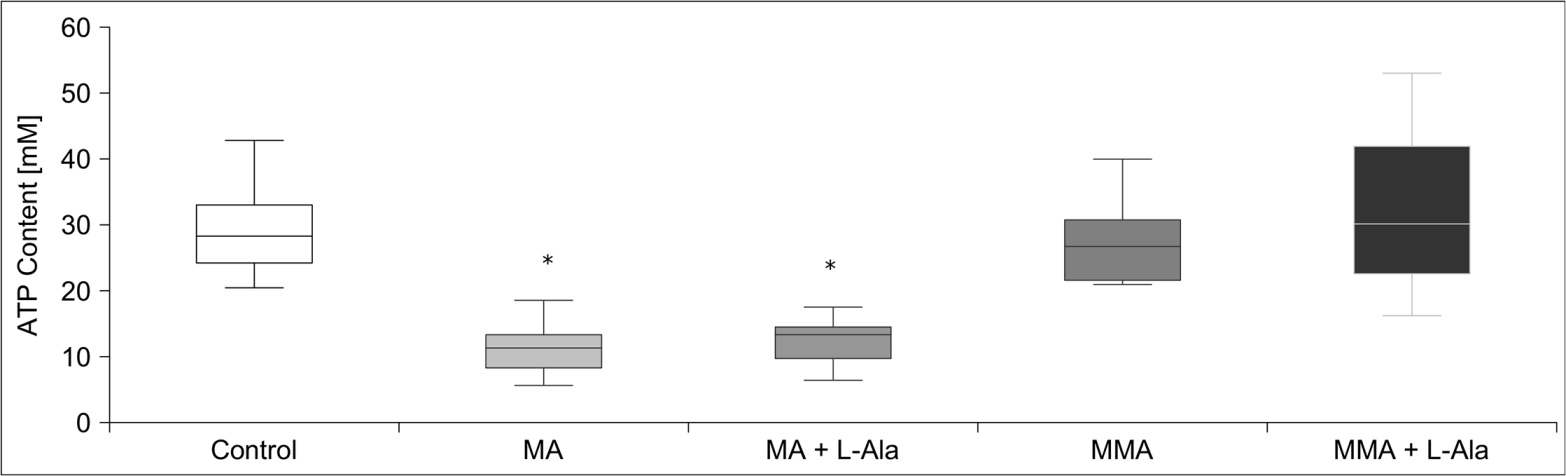
Effects of organic anion transporter inhibitors on MA cytotoxicity. The organic anion transporter inhibitor probenecid (P) blocked MA-induced LDH release, whereas succinic acid, *p*-aminohippuric acid (PAH) and taurocholic acid were ineffective. Data are presented as percent of untreated control of n = 4 independent experiments.

In line with MA-induced cell death, MA treatment caused a profound ATP loss already 6 h after treatment **(Fig 6)**. Though co-application of L-alanine abolished MA-induced cell death and LDH release, it did not restore or even affect cellular ATP content. The same effect was reproduced with other amino acids such as glycine and L-glutamate (data not shown).

**Fig 7:**
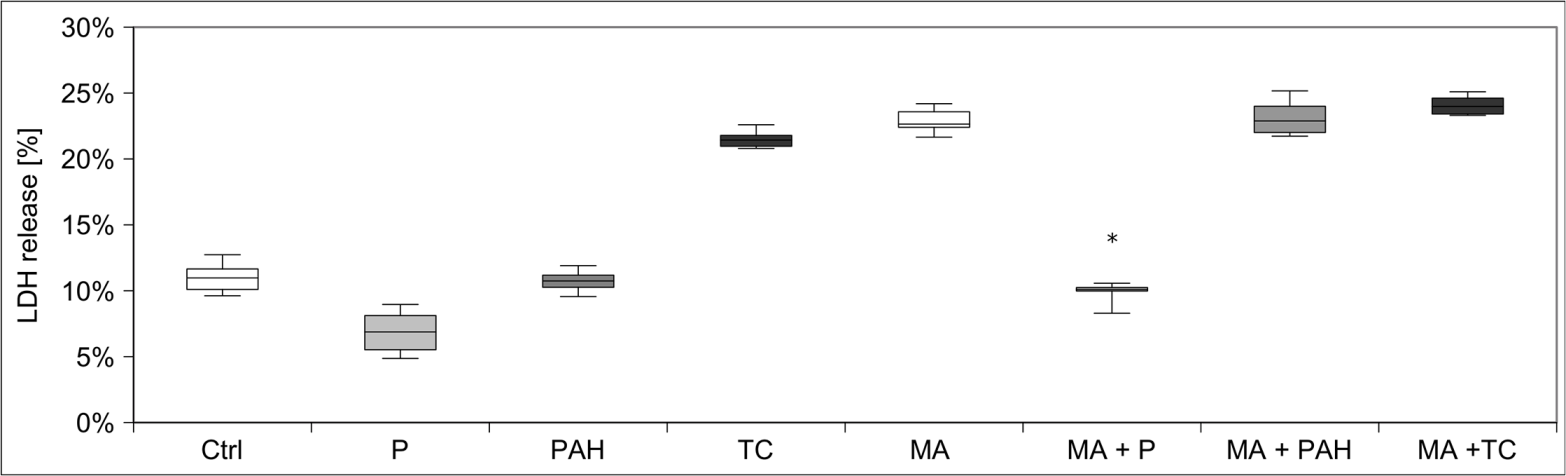
ATP levels in hPTECs before and after MA treatment. hPTECs showed a decreased ATP content 6h after treatment that was not affected by rescuing amino acids. In line with cell vitality experiments, MMA did not change cellular ATP levels. Data are presented as percent of untreated control of n = 4 independent experiments.

MA transport has been suggested to occur via sodium-dependent dicarboxylate transporters (NaC) [25]. Further, organic anion transporters (OAT) couple influx of monocarboxylic acids to sodium-dependent efflux of dicarboxylic acid and vice versa. Therefore, we tested the protective effect of blocking transport via NaC and OAT. Reducing sodium levels in incubation medium only mildly reduced MA-induced LDH release (treatment with MA and Na^+^: 25% ± 1%; treatment with MA without Na^+^: 20% ± 2%). Next, we tested whether co-application of the OAT substrates probenecid, *p*-aminohippuric acid, taurocholic acid (each 2 mM, for 24 h) reduced MA toxicity. Probenecid completely prevented MA-induced LDH release, whereas *p*-aminohippuric acid and taurocholic acid were ineffective **(Fig 7)**. Thus the uptake of MA into the cell occurs through probenecid sensitive OATs.

### Maleic acid impairs cellular energy homeostasis

**Fig 8:**
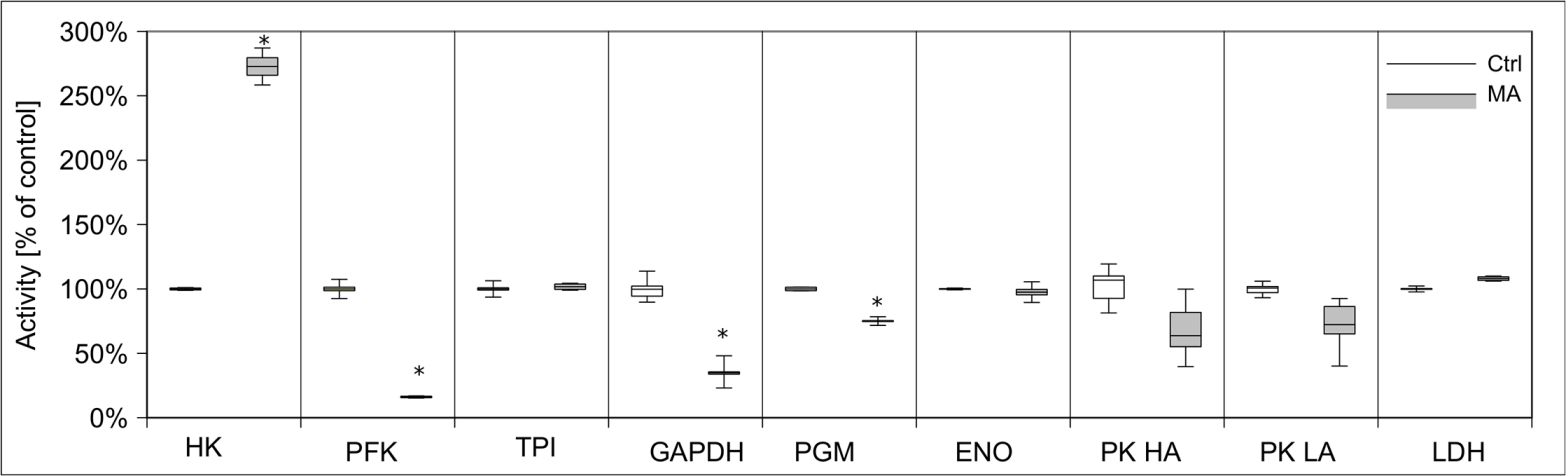
Effects of MA treatment on the enzymes of glycolysis. We analyzed activities of enzymes of glycolysis in hPTECs that were incubated for 24h with 21 mM MA. In treated cells, Hexokinase (HK) activity was increased, while phosphofructokinase (PFK), glyceraldehyde-3-phosphate dehydrogenase activity (GAPDH) and phosphoglycerate mutase (PGM) were reduced. Data are presented as percent of untreated control expressed of n = 3 independent experiments.

**Fig 9:**
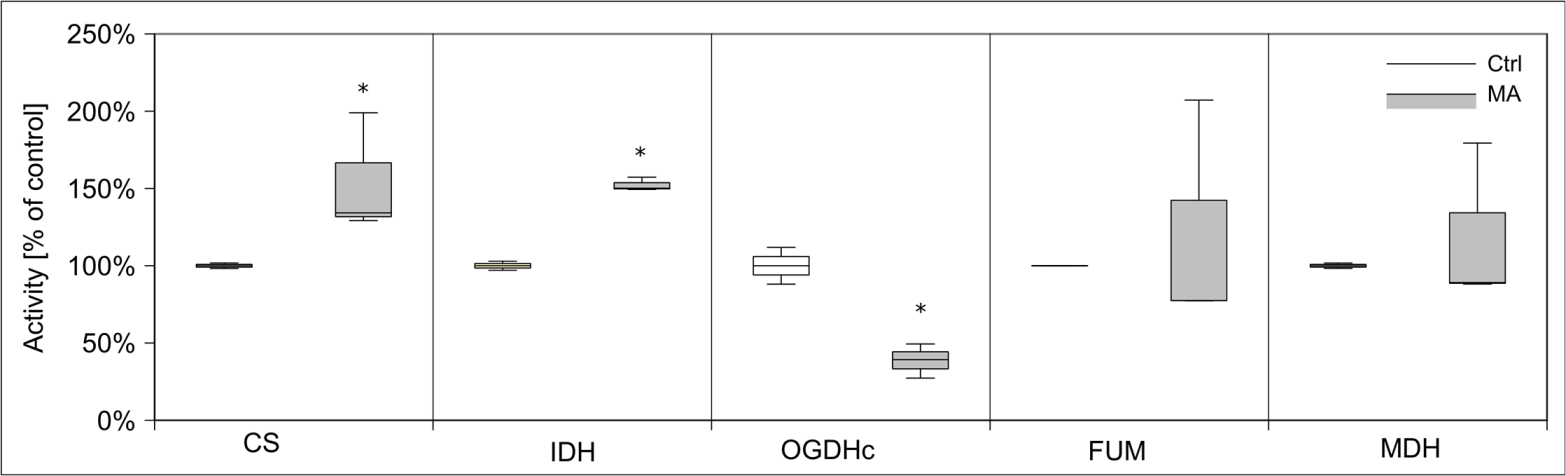
Effects of MA treatment on the citric acid cycle element activities. We analyzed activities of enzymes of citric acid cycle in hPTECs that were incubated for 24h with 21 mM MA. Activities of triosephosphate isomerase (TPI), enolase (ENO), high and low affinity pyruvate kinase (PK HA/LA), and lactate dehydrogenase (LDH) were unchanged. Moreover, enzymatic activity of the citric acid cycle protein 2-oxoglutarate dehydrogenase complex was diminished by MA treatment, whereas citrate synthase andisocitrate dehydrogenase (IDH) activities were increased. Activities of fumarase (FUM) and malate dehydrogenase (MDH) were not affected significantly. Data are presented as percent of untreated control expressed of n = 3 independent experiments.

**Fig 10:**
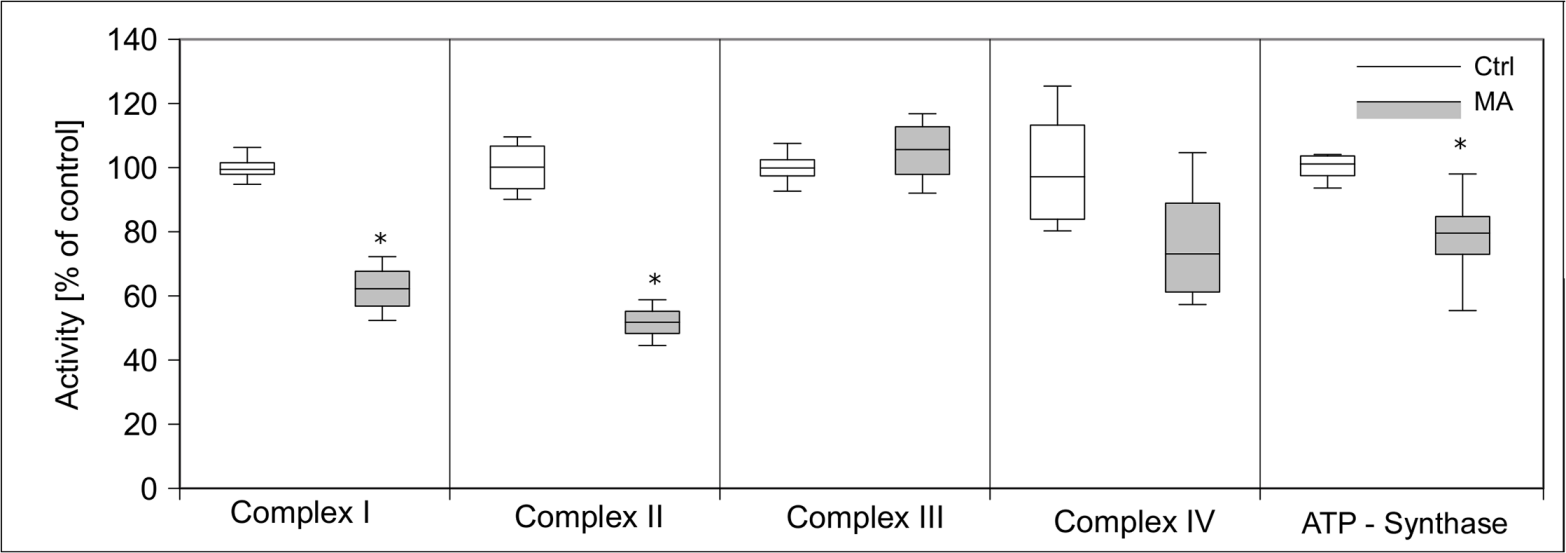
Effects of MA treatment on the respiratory chain complex activities. We analyzed activities of enzymes of respiratory chain in hPTECs that were incubated for 24h with 21 mM MA. Activities of respiratory chain complexes I and II were reduced by MA treatment, whereas ATP synthase was mildly and complex III and IV remained unaffected. Data are presented as percent of untreated control expressed of n = 3 independent experiments.

**Fig 11:**
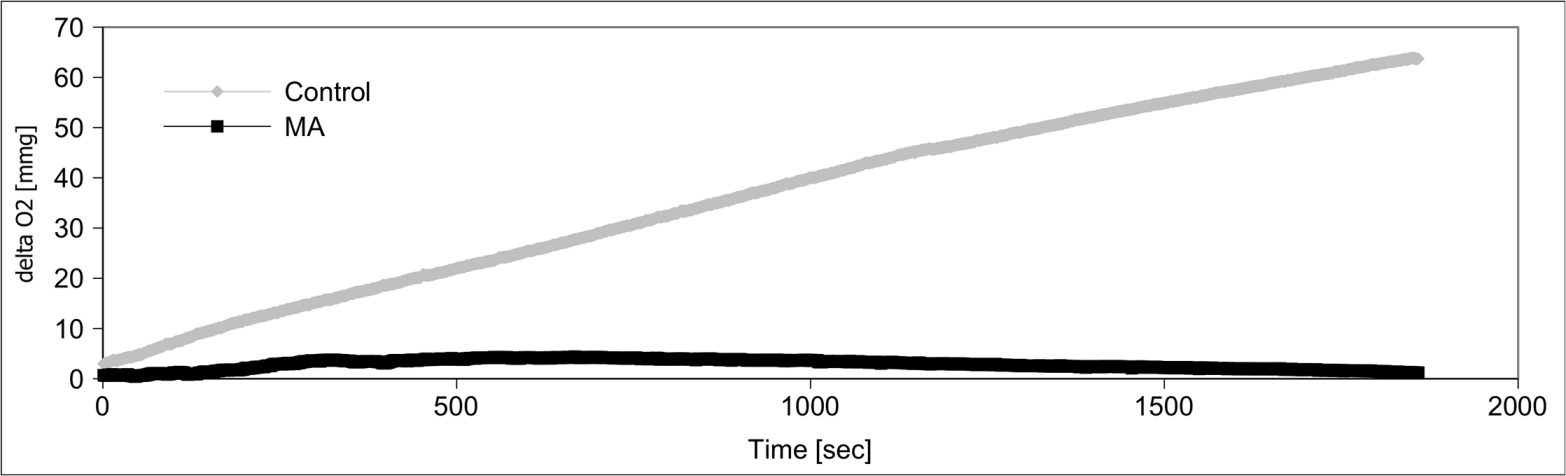
Effect of MA treatment on the mitochondrial oxygen consumption. As a measure of overall respiratory chain activity, we assessed mitochondrial oxygen consumption. hPTECs treated for 6h with MA did not reveal any NaCN-sensitive mitochondrial respiration. This figure depicts exemplary results from one experiment.

**Fig 12:**
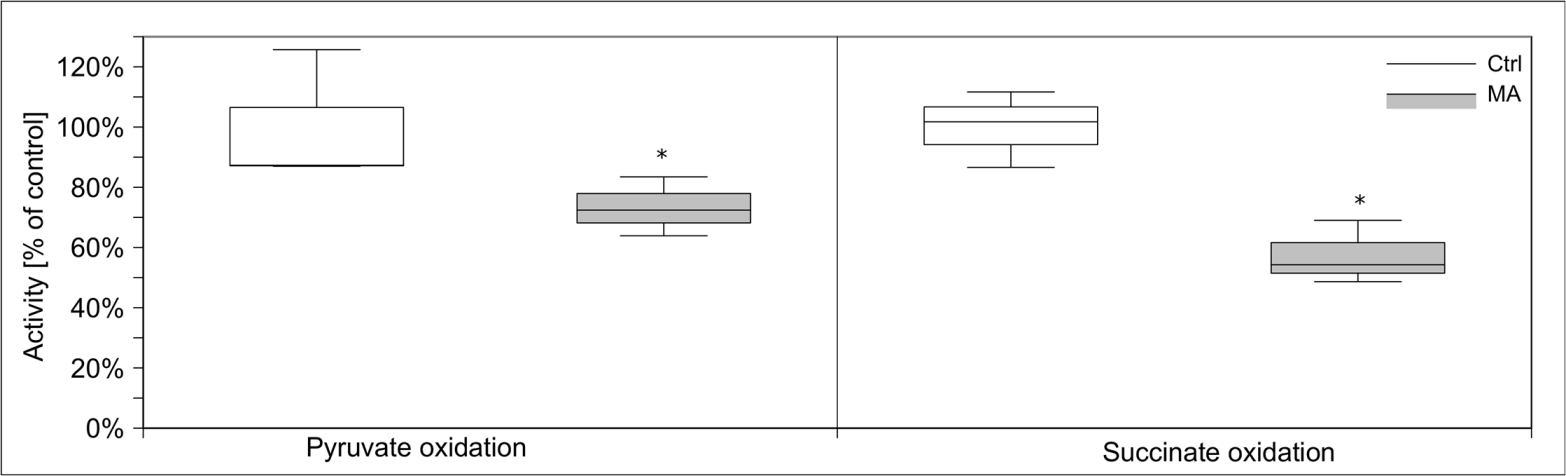
Effect of MA treatment on pyruvate and succinate oxidation. To estimate overall mitochondrial function we investigate pyruvate and succinate oxidation. CO_2_ production rates on both substrates were reduced after MA treatment (*12*). Data are presented as percent of untreated control expressed of n = 3 independent experiments.

Since MA caused a dramatic decrease of cellular ATP content, we tested its impact on cellular energy homeostasis, in particular glycolysis, respiratory chain, pyruvate dehydrogenase complex, and citric acid cycle. We analyzed the activity of those enzymes after 24 h of MA (21 mM) treatment in subcellular fractions compared to untreated control cells. Hexokinase activity increased more than two-fold by MA treatment, while phosphofructokinase activity decreased to 20% of its control level **(Fig 8)**. Glyceraldehyde-3-phosphate dehydrogenase activity was reduced by 65%. Activities of other tested glycolytic enzymes have not been altered. Next, we tested enzymatic activities of the citric acid cycle proteins citrate synthase, 2-oxoglutarate dehydrogenase complex, isocitrate dehydrogenase, fumarase, and malate dehydrogenase after MA treatment. MA strongly decreased 2-oxoglutarate dehydrogenase complex activity, but increased the activities of citrate synthase and isocitrate dehydrogenase. Activities of other citric acid cycle enzymes remained unchanged **(Fig 9)**. The activities of respiratory chain complexes I and II as well as ATP synthase activity were reduced by MA, whereas complex III and IV were not affected **(Fig 10)**. Next, we assessed mitochondrial oxygen consumption using an oxygen electrode. Already after 6 h, MA-treated hPTECs showed a complete loss of mitochondrial respiration **(Fig 11)**. In contrast, application of MA to hPTECs did not directly change mitochondrial respiration within 1 h of incubation (data not shown). To estimate overall mitochondrial function we compared pyruvate and succinate oxidation in treated and untreated cells. MA treated cells showed decreased CO_2_ production rates on both substrates **(Fig 12)**. Overall MA treatment disturbs the energy metabolism in an enzymatic level.

### Intracellular metabolism of maleic acid

To gain further insight in the mechanism of MA toxicity, we first tested whether MA is metabolized by hPTECs. After 24 h of treatment medium concentrations of MA were virtually unchanged indicating that it is not effectively degraded (MA detection via GC/MS, data not shown). Moreover, application of rescuing amino acids did not affect intracellular or extracellular MA concentrations. Since the addition of L-alanine and structural similar amino acids prevented MA toxicity, we studied the intracellular amino acid pool **(Table 1)**. MA treatment caused a general decrease in all tested amino acids being most pronounced for L-glutamate, L-tryptophan and L-proline (more than 5 times). The applied rescuing amino acids replenished their own intracellular concentrations. However, this was also the case when L-phenylalanine was added that had even shown a mild toxic effect on hPTECs. In concert with unchanged ATP levels this finding stresses that the rescuing effect is not based on anaplerotic pathways. It has been speculated that MA nephrotoxicity can at least partially be attributed to the formation of maleyl-CoA and depletion of the free CoA pool [12]. Mitochondrial beta-oxidation depends on activation of fatty acids by CoA ligation and depletion of this cofactor will impair fatty acid break down in mitochondria. If beta-oxidation is impaired cells export accumulating fatty acids as acylcarnitines. Therefore, cellular acylcarnitine status is a good indicator for beta-oxidation defects and also used in newborn screening. Metabolic profiling after MA treatment revealed no consistent pattern of changes in the acylcarnitine profile that would indicate disturbance of mitochondrial beta-oxidation. **(Table 2)**

**Table 1:** Amino acid profile in hPTEC with and without MA loading.

	Ala	Val	Met	Phe	Tyr	Asp	Glu	Trp	Pro	His	Thr	Gly	Orn	Arg	Cit
**Ctrl**	54.77 ± 5.3	49.35 ± 14.58	14.33 ± 4.43	44.08 ± 5.52	33.01 ± 0.44	50.00 ±11.08	434.21 ± 19.67	48.47 ± 3.68	223.10 ± 75.05	110.73 ± 16.07	53.86 ± 5.93	134.35 ± 16.58	12.11 ± 1.15	98.95 ± 7.74	6.62 ± 2.22
**MA**	14.41* ± 0.37	26.52* ± 0.47	3.29* ± 0.49	15.41* ± 1.25	8.69* ± 0.45	17.00* ± 3.01	36.00* ± 1.74	5.69* ± 0.04	25.11* ± 3.08	35.15* ± 15.54	14.79* ± 1.84	36.72* ± 1.83	7.76* ± 0.03	38.93* ± 1.44	3.33* ± 0.31
**MA + Gly**	11.56* ± 0.11	31.33* ± 2.85	3.36* ± 0.49	13.00* ± 1.15	12.22* ± 2.26	12.11* ± 0.46	68.99* ± 10.39	11.66* ± 0.44	23.14* ± 4.49	32.39* ± 3.99	14.27* ± 0.70	N/A	6.11* ± 1.14	37.57* ± 4.48	3.12* ± 0.02
**MA + Ala**	N/A	28.38* ± 1.05	3.08* ± 0.10	11.86* ± 0.68	11.79* ± 0.19	17.41* ± 1.48	46.00* ± 3.79	7.83* ± 1.39	25.86* ± 0.88	38.25* ± 7.40	18.54* ± 2.87	36.27* ± 0.81	7.73* ± 0.76	38.78* ± 0.22	3.52* ± 0.54
**MA + Phe**	20.15* ± 3.92	27.13* ± 3.51	3.93* ± 1.13	N/A	8.53* ± 0.06	18.98* ± 5.08	25.77* ± 0.88	3.74* ± 0.85	22.58* ± 6.94	39.81* ± 3.25	16.86* ± 1.97	37.26* ± 1.41	22.37 ± 6.94	34.67* ± 0.66	4.12* ± 0.33

Data are presented in nmol/mg protein expressed as mean of n = 3 experiments ± SD.

*p<0.05, rANOVA

**Table 2:** Acylcarnitine profile in hPTEC with and without MA loading.

	C0	C2	C3	C4	C5	C6	C8	C10	C12	C14	C14OH	C16	C16OH	C18	C18OH
**Ctrl**	1.52 ± 0.13	2.59 ± 0.19	0.17 ± 0.04	0.09 ± 0.04	0.06 ± 0.02	0.03 ± 0.00	0.02 ± 0.01	0.01 ± 0.00	0.01 ± 0.00	0.01 ± 0.01	0.02 ± 0.01	0.04 ± 0.01	0.01 ± 0.01	0.03 ± 0.01	0.01 ± 0.01
**MA**	0.52* ± 0.14	0.22* ± 0.03	0.03* ± 0.01	0.01 ± 0.00	0.01 ± 0.01	0.03 ± 0.01	0.02 ± 0.00	0.01 ± 0.01	0.02 ± 0.01	0.03 ± 0.01	0.02 ± 0.01	0.22* ± 0.01	0.02 ± 0.00	0.08* ± 0.04	0.02 ± 0.02
**MA + Gly**	0.33* ± 0.01	0.30* ± 0.08	0.03* ± 0.01	0.01 ± 0.01	0.02 ± 0.01	0.03 ± 0.01	0.02 ± 0.01	0.01 ± 0.00	0.01 ± 0.01	0.03 ± 0.01	0.01 ± 0.00	0.18* ± 0.04	0.03 ± 0.02	0.08* ± 0.00	0.00 ± 0.00
**MA + Ala**	0.31* ± 0.03	0.22* ± 0.01	0.02* ± 0.01	0.01 ± 0.01	0.01 ± 0.01	0.02 ± 0.01	0.02 ± 0.01	0.01 ± 0.01	0.01 ± 0.00	0.02 ± 0.00	0.02 ± 0.01	0.22* ± 0.01	0.02 ± 0.03	0.08* ± 0.00	0.03 ± 0.01
**MA + Phe**	0.50* ± 0.21	0.24* ± 0.04	0.02* ± 0.01	0.02 ± 0.01	0.01 ± 0.00	0.02 ± 0.00	0.01 ± 0.00	0.02 ± 0.01	0.01 ± 0.01	0.02 ± 0.01	0.02 ± 0.00	0.26* ± 0.01	0.02 ± 0.00	0.10* ± 0.03	0.02 ± 0.01

Data are presented in nmol/mg protein expressed as mean of n = 3 experiments ± SD.

*p<0.05, rANOVA

### Maleic acid disturbs cellular calcium homeostasis

**Fig 13:**
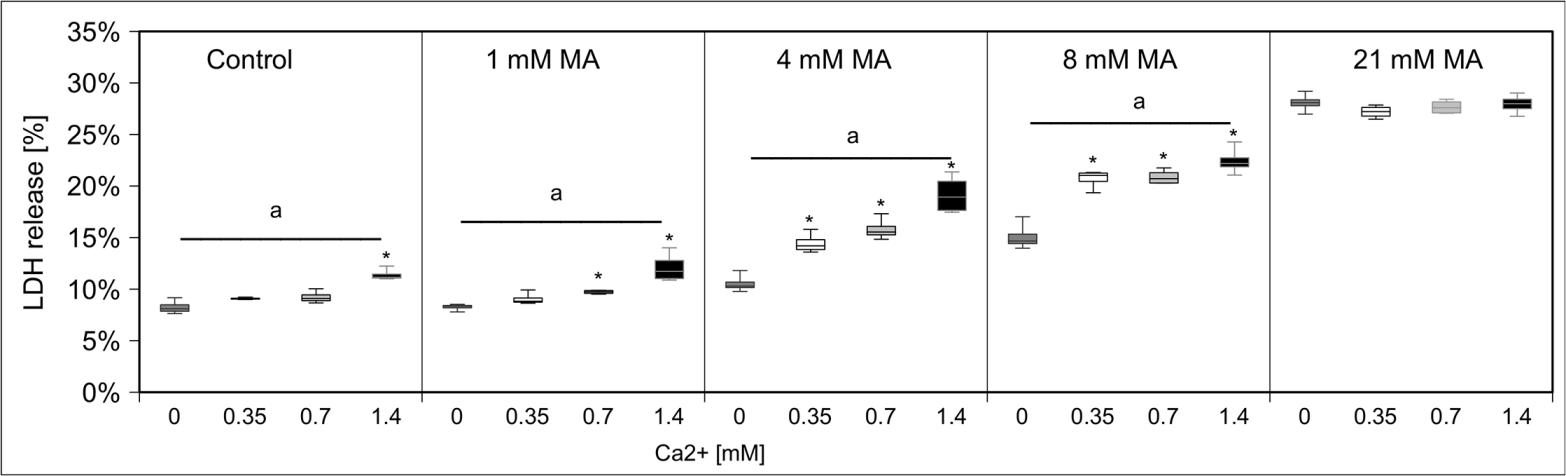
Effect of calcium concentration on MA-induced LDH release. Decreasing calcium concentrations in treatment buffer (0, 0.35, 0.7, 1.4 mM) reduced MA induced LDH release except for the highest applied MA concentration. Data are presented as percent of untreated control of n = 5 independent experiments.

**Fig 14:**
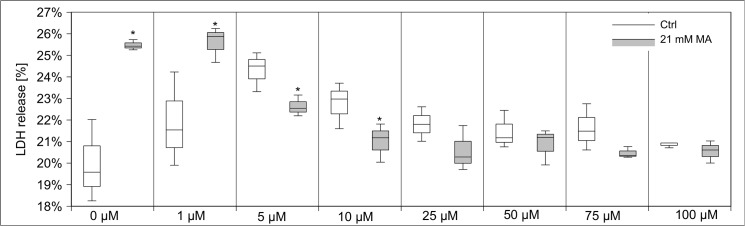
BAPTA-AM reduces MA-induced LDH release. The selective chelator of intracellular Ca^2+^ stores BAPTA-AM mimicked the previous approach (Fig 13). Data are presented as percent of untreated control of n = 5 independent experiments.

**Fig 15:**
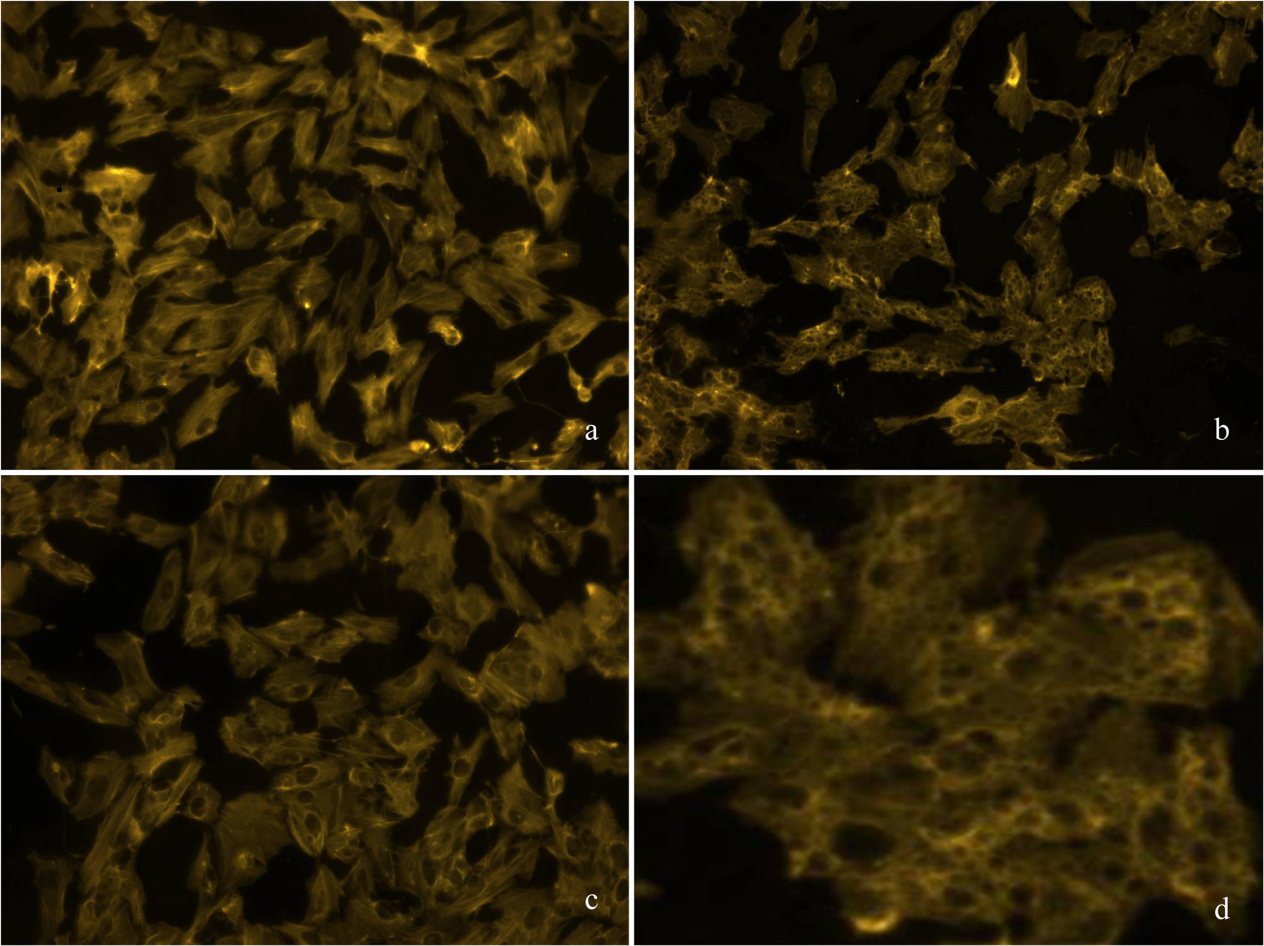
a-d. MA leads to vacuole formation in hPTECs. Staining of actin revealed intensive vacuole formation in MA-loaded (21 mM) hPTECs that could be prevented by co-incubation with rescuing amino acids. Exemplarily results for amino acid L-glutamate are shown.

**Fig 16:**
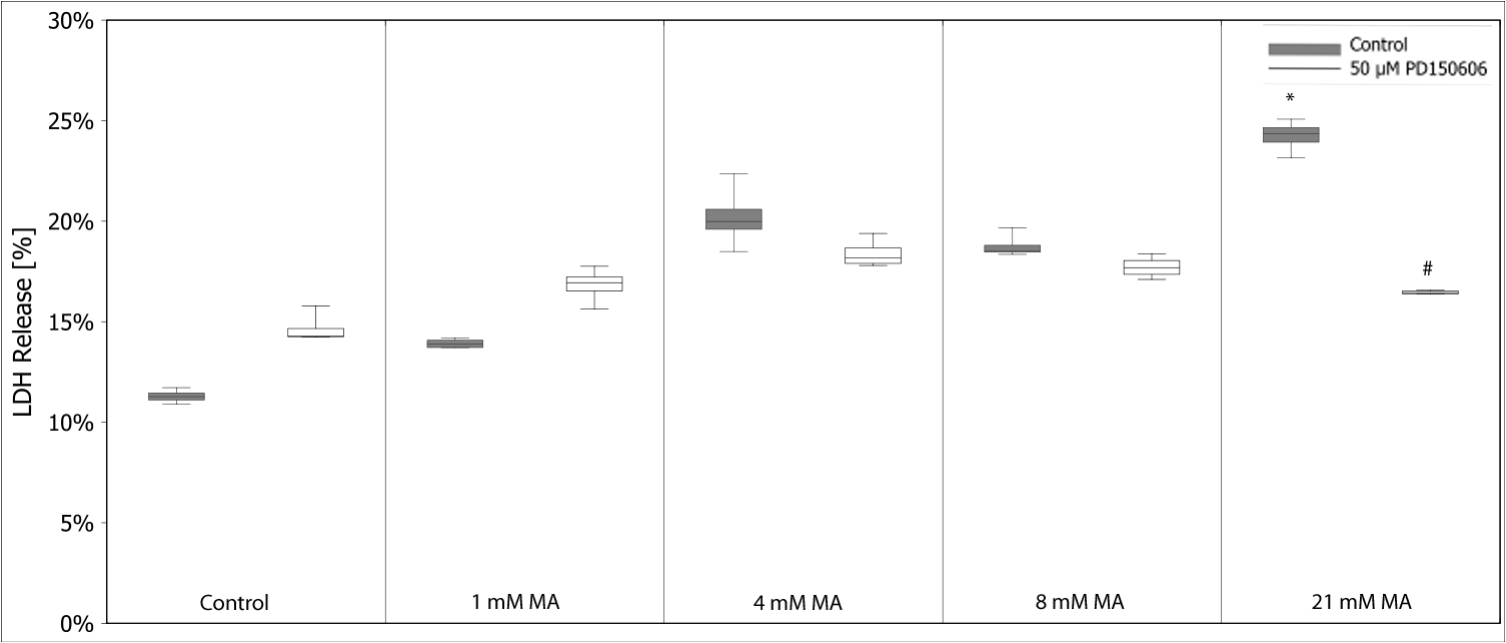
MA toxicity activates calpain pathways. MA treatment activated calcium-dependent calpain proteases as indicated by the reduction of MA-induced LDH release by the inhibitor PD 150606 (50 µM). Data are presented as percent of MA treated control cells of n = 5 independent experiments.

Calcium has been linked to MA toxicity [12]. Therefore, we modulated calcium homeostasis by varying calcium levels in buffer, adding calcium transport inhibitors, and intracellular calcium chelators. Varying calcium concentrations in treatment buffer (0, 0.35, 0.7 and 1.4 mM CaCl_2_) demonstrated that MA-induced toxicity concomitantly increased with calcium concentrations–except for the highest applied MA concentration **(Fig 13)**. Next, we inhibited cellular calcium uptake by the calcium channel blocker nifedipine (0–250 µM) **(S1 Fig)**. In line with the previous experiment, MA toxicity was strongly reduced by nifedipine. Applying BAPTA-AM, a selective chelator of intracellular Ca^2+^ stores, gave the same result reducing toxic effects of MA, though to a lesser extent due to its own toxicity **(Fig 14)**. It has previously been demonstrated that MA can act as calcium chelator and augment calcium levels in the ER stores [26]. Moreover, MA can influence and disturb ER membrane transport processes [13]. Several studies highlight that ER stress, disturbance of calcium homeostasis and ATP depletion go along with cytoplasmic vacuolization. Staining of actin skeleton showed intensive vacuole formation of hPTECs stressed with MA **(Fig 15A–15D)**. Of note, these changes were fully blocked by the addition of rescuing amino acids. Interaction of the calpain system and ER has been identified as a central mechanism in renal damage and cell death due to cytotoxic substances [27]. PD 150606 is a selective uncompetitive calpain inhibitor of the calcium-dependent cysteine protease calpain. Co-application of PD 150606 (50 µM) and MA strongly reduced MA-induced cytotoxicity in hPTECs **(Fig 16)**. Thus MA unfolds its cytotoxic effects by disturbing cellular Ca^2+^ homeostasis.

### Maleic acid toxicity is dependent on chloride ions

**Fig 17:**
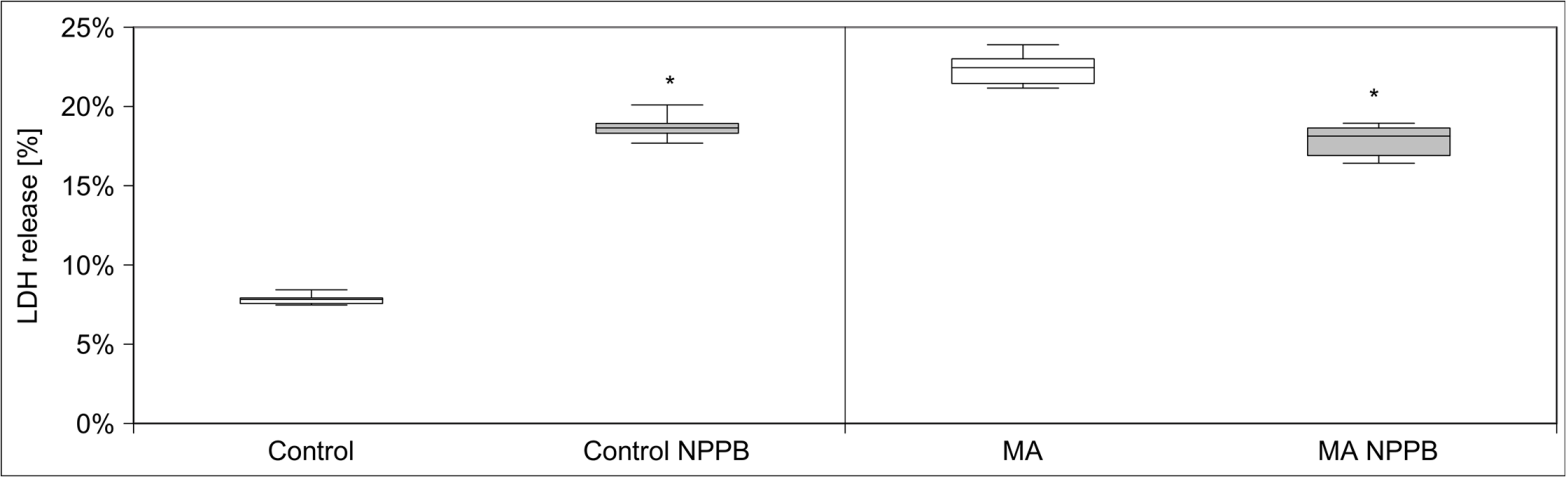
Effect of chloride channel blocker NPPB on MA mediated LDH release. The chloride channel blocker NPPB (10 µM) decreased MA induced LDH release to the corresponding control level. Data are presented as percent of untreated control of n = 6 independent experiments.

**Fig 18:**
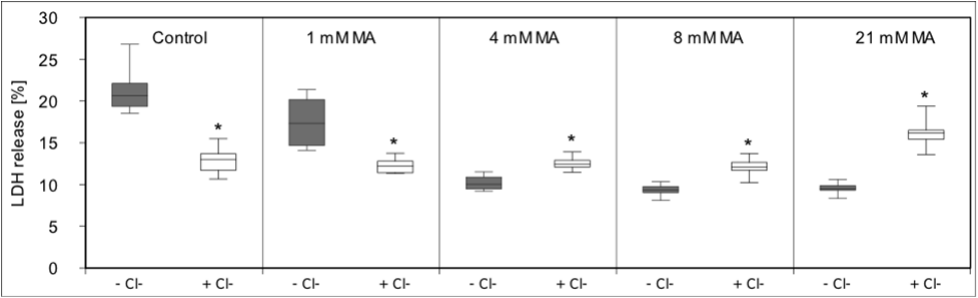
Effect of chloride ions on MA mediated LDH release. Incubation of hPTEC in chloride-free KRB resulted in high LDH release rates. Strikingly, this effect was reduced by the addition of MA. Data are presented as percent of untreated control of n = 6 independent experiments.

Waters and Schnellmann [28] showed that the influx of chloride ion is a late event in toxin-induced renal cell death following calpain activation. Therefore we tested the effect of chloride ions in the medium and the chloride channel blocker 5-Nitro-2-(3-phenylpropylamino) benzoic acid (5-NPPB) on maleic toxicity **(Fig 17)**. Even in small concentrations 5-NPPB was toxic to hPTECs, however in the smallest concentrations used (10 µM), it used MA-induced LDH release to the corresponding control level. Chloride-free KRB caused a high LDH release rate in hPTECs **(Fig 18)**. Interestingly, the lack of chloride ions was counterbalanced by the addition of MA. At the highest used MA concentration (21 mM), hPTECs in chloride-free KRB showed LDH release rate similar to control conditions.

## Discussion

MA application is a well-established model to induce Fanconi syndrome in different rodents. However, the underlying pathomechanism is yet under debate. Studies link MA-induced toxicity to impairment of tubular transport by inhibition of Na^+^-K^+^-ATPases [7, 8] and mitochondrial dysfunction [9–11]. It has also been suggested that the formation of maleyl-CoA imbalances fatty acid metabolism and, thereby, damage proximal tubule [12]. Moreover, impairment of endoplasmic reticulum membrane transport [13] and impairment of calcium homeostasis by MA were found [12]. To identify MA-induced mechanisms, we performed parallel experiments with MMA, a dicarboxylic acid accumulating in methylmalonic acidurias. We have chosen MMA for the following reasons, (1) it is structurally similar to MA (e.g. similar cellular uptake mechanism and detoxification mechanism such as CoA conjugation), (2) it is very slowly degraded in metabolically active cells therefore allowing to differentiate between unspecific (e.g. osmotic pressure) and MA-specific effects and (3) the renal phenotype of MA-induced toxicity (Fanconi syndrome) and methylmalonic acidurias (interstitial nephritis) differs [29]. In line with this notion, exposure to MA induced time- and concentration-dependent cytotoxicity in hPTECs with a complex pathomechanism, whereas all tested biochemical and bioenergetic parameters of hPTECs remained unchanged by methymalonic acid. This finding underlines the specificity of the MA-induced pathology.

MA-induced cell damage was associated with a dramatic loss of cellular ATP-pool. In line with this finding, energy homeostasis was severely disturbed on the level of glycolysis, citric acid cycle, and respiratory chain showing strongly reduced activities of PFK, GAPDH, OGDHc, complex I and II. Moreover, reduced pyruvate and succinate oxidation rates as well as undetectable NaCN-sensitive mitochondrial respiration provide proof for MA-induced mitochondrial dysfunction. These effects were not mediated by direct inhibitory effects of MA on bioenergetic proteins.

Cellular uptake of MA may occur via organic anion transporter 4 (OAT4) or sodium-dependent dicarboxylate transporter 1 (NaC1) that are both expressed at the apical site of proximal tubule [30]. MA-induced LDH release was reduced in the absence of sodium, but blocked by the organic anion transport inhibitor probenecid. Both findings indicate that MA is transported via OAT4 that is highly susceptible to probenecid and secondary dependent on sodium gradient.

Strikingly, co-incubation with single amino acids (L-alanine > L-glutamate > L-glycine > D-alanine > β-alanine) reduced or even prevented MA-induced toxicity. There was however no significant difference between treatment with L-alanine and L-glutamate. This effect was not based on replenishment of intracellular ATP pool excluding stimulation of anaplerotic pathways. Further, amino acid profiling showed a considerable decline in intracellular amino acid concentrations following MA treatment, but co-incubation with glycine, L-alanine or L-phenylalanine did not increase these concentrations. Moreover, rescuing amino acids also blocked MA-induced cytosolic vacuolization. The cytoprotective effect of glycine and structural similar amino acids especially for proximal tubule cells against toxic agents has been described before, but the underlying mechanism is yet not elucidated [31, 32]. Why treatment with L-phenylalanine and L-lysine enhances LDH release, remains to be elucidated.

We could directly link MA toxicity to disturbance of calcium homeostasis. Decreasing calcium concentrations in the incubation medium, the calcium channel blocker Nifedipine, as well as the intracellular calcium chelator BAPTA-AM reduced MA mediated LDH release. Moreover, the protective effect of PD150606 connects MA toxicity to activation of the calcium dependent calpain proteases that is typically found in toxicant induced proximal tubule damage. PD150606 is an inhibitor of calpain proteases found in the apoptosis pathways. Moreover, we found that MA concentration-dependently induces apoptosis further linking its toxic effect to calpain proteases-mediated apoptosis. PD150606 treatment per se induces a slight induction of cell death. Thus the inhibition of MA-induced apoptotic cell death for lower MA levels (4–8 mM) appears less effective due to increased basal cell death level. Nevertheless, the rescuing effect of PD150606, prevention of apoptosis, is still significant.

Previous studies have shown that MA increases calcium levels in endoplasmic reticulum by acting as a chelator [26] and disturbs membrane transport processes of this organelle [13]. Further, several studies revealed that activation of calpain proteases occurs due to calcium overload and endoplasmic reticulum stress [27]. Based on these findings and the results of the current study, it seems likely that MA toxicity is based on calcium overload and endoplasmic reticulum stress resulting in activation of calpain proteases and cell death. We further aimed to localize the observed dysfunction of cellular energy homeostasis in this scenario. As shown by our study, MA does not have a direct effect on any of the investigated proteins of glycolysis, citric acid cycle, or respiratory chain. In contrast the observed changes can be attributed to calcium overload. Besides endoplasmic reticulum mitochondria are cellular calcium stores. Mitochondrial calcium overload has deleterious effects on this organelle leading to complete mitochondrial dysfunction as seen after MA treatment in our experiments. It has been discussed that production of reactive oxygen species (ROS) is another result of this effect [33]. Within glycolysis MA application had the strongest impact on PFK and to a lesser extent on GAPDH activity. The first protein can exist in nearly inactive dimeric or active tetrameric form. Previous studies have shown that when activated calmodulin binds to both high affinity and both low affinity binding sites of PFK, the tetramer dissociates to the inactive dimeric form [34]. Further, GAPDH is highly susceptible to ROS leading to oxidative modification of its active site and, subsequently, to inactivation of its catalytic activity [35]. Intensive ROS production is a direct consequence of mitochondrial dysfunction.

In conclusion, our study shows that MA but not MMA exerts toxic effects on hPTECs. MA-induced toxicity is mediated by disturbed calcium homeostasis, in particular ER calcium overload and activation of calpain proteases resulting in apoptosis, and is accompanied by disturbed energy homeostasis. Our study therefore provides a revised mechanism for MA-induced renal cell damage.

## Supporting Information

S1 Fig Nifedipin reduces maleic acid toxicity. Inhibition of cellular calcium uptake by the calcium channel blocker nifedipin (0–250µM) diminished MA toxicity.
(TIF)

S1 Tables Each table shows the corresponding p-values of the compared groups. An “*a*”, “#” or “*” indicates that the results are significantly different.
(PDF)
