# Supplementary figures and images for: Maleic Acid – but Not Structurally Related Methylmalonic Acid – Interrupts Energy Metabolism by Impaired Calcium Homeostasis
Source: PLoS One. 2015 Jun 18;10(6):e0128770. doi: 10.1371/journal.pone.0128770 (PMC4473014; doi:10.1371/journal.pone.0128770)

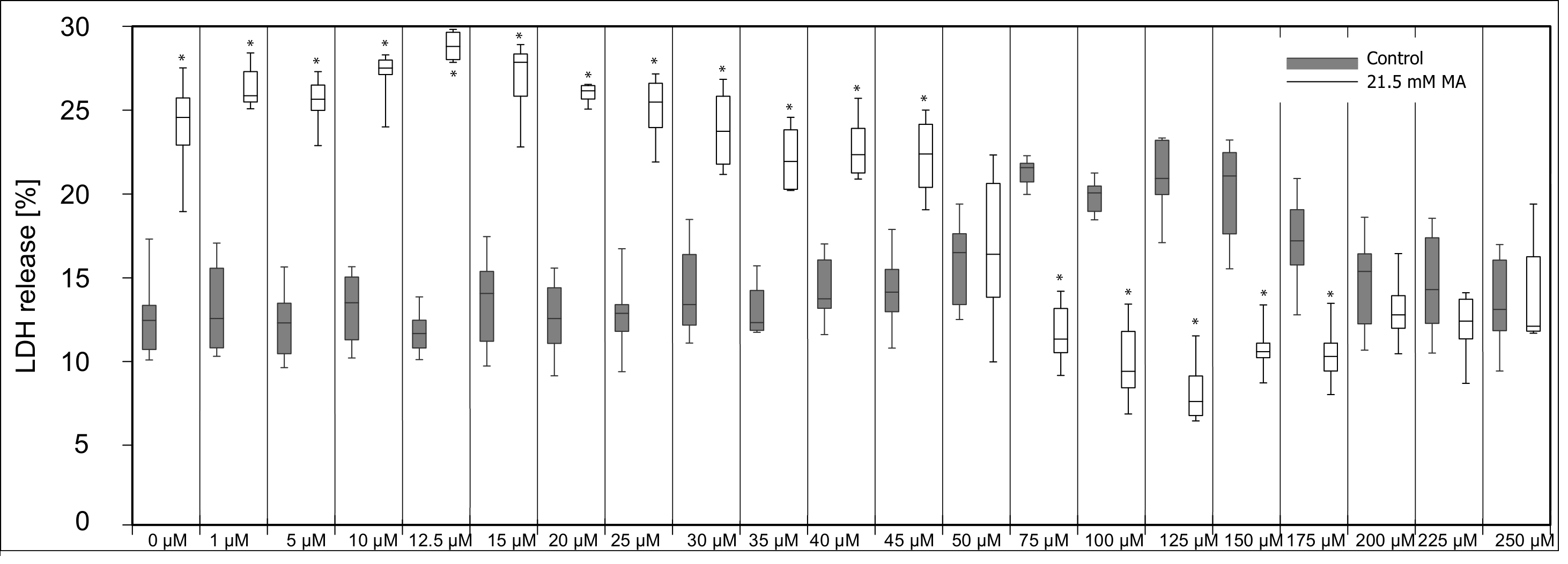

Supplement: S1 Fig — Inhibition of cellular calcium uptake by the calcium channel blocker nifedipin (0–250µM) diminished MA toxicity. (TIF) [file pone.0128770.s001.tif]
