# Supplementary material for: Maleic Acid – but Not Structurally Related Methylmalonic Acid – Interrupts Energy Metabolism by Impaired Calcium Homeostasis
Source: PLoS One. 2015 Jun 18;10(6):e0128770. doi: 10.1371/journal.pone.0128770 (PMC4473014; doi:10.1371/journal.pone.0128770)
Supplement: S1 Tables — An “a”, “#” or “*” indicates that the results are significantly different. (PDF) [file pone.0128770.s002.pdf]

Supplementary Tables

Figure 1a:

	F	P-Value	F crit
**rANOVA for 1 h:**	0.061614	0.992711	2.605975
**rANOVA for 4 h:**	0.021318	0.999075	2.605975
**rANOVA for 8 h:**	0.209471	0.931668	2.605975
**rANOVA for 12 h:**	25.54125	3.13E-05[a]	3.47805
**rANOVA for 18 h:**	12.32222	0.000704[a]	3.47805
**rANOVA for 24 h:**	21.81331	1.6E-08[a]	2.689628

vs. (p-values for t-test)	1 mM	4 mM	8 mM	21 mM
**Control (1 h)**	0.4052	0.3819	0.4932	0.3451
**Control (4 h)**	0.4611	0.4218	0.4754	0.4410
**Control (8 h)**	0.4721	0.3925	0.4521	0.2270
**Control (12 h)**	0.0300	0.0470	0.0008*	0.0019*
**Control (18 h)**	0.2437	0.0011*	0.0009*	0.0077*
**Control (24 h)**	0.3408	0.0011*	0.0001*	0.0000*

[a]p<0.005 rANOVA. *p<0.005, Student's t-test

Figure 1b:

vs. (p-values for t-test)	Control	1 mM MA	4 mM MA	8 mM MA	21 mM MA
**Control**	0.005355399				
**1 mM MMA**		3.41686E-05*			
**4 mM MMA**			3.88342E-07*		
**8 mM MMA**				3.97323E-07*	
**21 mM MMA**					3.29179E-07*

vs. (p-values for t-test)	Control
**1 mM MA**	0.00315706
**4 mM MA**	2.48318E-07*
**8 mM MA**	7.67054E-08*
**21 mM MA**	1.03602E-09*

vs. (p-values for t-test)	Control
**1 mM MMA**	0.306784102
**4 mM MMA**	0.052550104
**8 mM MMA**	0.003623686
**21 mM MMA**	5.06838E-06*

*p<0.001, Student's t-test

Figure 1d:

vs. (p-values for t-test)	Control
**1 mM MA**	0.15042708
**4 mM MA**	0.40348657
**8 mM MA**	0.00010413*

**21 mM MA**	1.2331E-05*

*p<0.001, Student's t-test

Figure 1e:

**vs. (p-values for t-test)**	**MA**	**L-Glu**
**Control**	8.5467E-20*	
**ß-Ala**	0.00081579*	
**D-Ala**	0.00196619*	
**L-Ala**	4.5879E-09*	0.08969426
**L-Arg**	0.00999084	
**L-Glu**	2.5828E-10*	
**Gly**	3.3997E-05*	
**L-Lys**	0.03771759	
**L-Pro**	0.12016754	
**L-Ser**	0.06214886	
**Tau**	0.33142781	
**Succ**	0.36519745	

*p<0.001, Student's t-test

Figure 1f:

**vs. (p-values for t-test)**	**Control**	**21.5 mM MA**
**21.5 mM MA**	4.34061E-15*	---
**21.5 mM MA + 5 mM L-Ala**	5.99002E-12*	0.198288107
**21.5 mM MMA**	0.313497545	1.79171E-08*
**21.5 mM MMA + 5 mM L-Ala**	4.34061E-15*	2.15239E-07*

*p<0.0001, Student's t-test

Figure 1g:

**vs. (p-values for t-test)**	**Ctrl**	**MA**
**P**	0.000308774	---
**PAH**	0.329873189	---
**TC**	1.81309E-09	---
**MA**	1.60581E-09*	---
**MA + P**	0.042345035	1.01924E-10*
**MA + PAH**	7.40637E-09	0.370647766
**MA +TC**	2.94881E-10	0.020183658

*p<0.001, Student's t-test

Figure 2a:

**p-values for t-test**	**Ctrl vs MA**

**HK**	1.61022E-12*
**PFK**	7.06258E-13*
**TPI**	0.196809436
**GAPDH**	1.57023E-06*
**PGM**	1.52788E-14*
**ENO**	0.101728814
**PK (HA)**	0.000206037
**PK (LA)**	0.000130428
**LDH**	2.41882E-06*

*p<0.0001, Student's t-test

Figure 2b:

p-values for t-test	Ctrl vs. MA
**CS**	0.03900*
**IDH**	0.00003*
**OGDHc**	0.00141*
**FUM**	0.32880
**MDH**	0.28327
**PDHc**	0.06489

*p<0.05, Student's t-test

Figure 2c:

p-values for t-test	Ctrl vs. MA
**Komplex I**	0.0001*
**Komplex II**	0.0001*
**Komplex III**	0.2305
**Komplex IV**	0.0900
**Komplex V**	0.0263*

*p<0.05, Student's t-test

Figure 2e:

p-values for t-test	Ctrl vs. MA
**Pyruvate Ox.**	0.0323*
**Succinate Ox.**	0.0054*

*p<0.05, Student's t-test

Figure 3a:

	F	P-Value	F crit
**rANOVA for Control (0 mM MA)**	25.2639256	1.797E-05[a]	3.49029482
**rANOVA for 1 mM MA:**	16.9799053	0.00012883[a]	3.49029482
**rANOVA for 4 mM MA:**	31.5233408	5.6817E-06[a]	3.49029482

**rANOVA for 8 mM MA:**	33.5154579	4.1071E-06[a]	3.49029482
**rANOVA for 21 mM MA:**	0.95332578	0.44593242	3.49029482

vs. (p-value for t-test)	0.35 mM Ca	0.7 mM Ca	1.4 mM Ca
**Control (0 mM MA)**	0.0234	0.0354	0.0002*
**Control (1 mM MA)**	0.0272	0.0001*	0.0010*
**Control (4 mM MA)**	0.0005*	0.0001*	0.0001*
**Control (8 mM MA)**	0.0002*	0.0001*	0.0001*
**Control (21 mM MA)**	0.0787	0.2466	0.4186

[a]p<0.01 rANOVA. *p<0.01, Student's t-test

Supplementary Figure 1:

p-values for t-test	Ctrl vs. 21 mM MA
**0 µM**	4.1338E-11*
**1 µM**	8.213E-07*
**5 µM**	1.879E-07*
**10 µM**	1.3297E-07*
**12.5 µM**	1.0425E-10*
**15 µM**	3.3949E-06*
**20 µM**	3.0051E-06*
**25 µM**	1.8793E-12*
**30 µM**	0.00043148*
**35 µM**	4.2881E-05*
**40 µM**	0.00014376*
**45 µM**	0.00010483*
**50 µM**	0.33942796
**75 µM**	3.1147E-07*
**100 µM**	2.7026E-06*
**125 µM**	3.3754E-06*
**150 µM**	4.0317E-05*
**175 µM**	0.00037878*
**200 µM**	0.15987062
**225 µM**	0.07433828
**250 µM**	0.37797037

*p<0.001, Student’s t-test

Figure 3b:

p-values for t-test	Ctrl vs. MA
**Control**	0.00386*
**1 µM BAPTA-AM**	0.02567*
**5 µM BAPTA-AM**	0.02428*
**10 µM BAPTA-AM**	0.04794*
**25 µM BAPTA-AM**	0.09055

**50 µM BAPTA-AM**	0.22301
**75 µM BAPTA-AM**	0.07368
**100 µM BAPTA-AM**	0.18252

*p<0.05, Student’s t-test

Figure 3g:

p-values for t-test	Ctrl vs. 50 µM PD150606
**0 mM MA**	9.99523E-05
**0.86 mM MA**	0.000360086
**4.3 mM MA**	0.041800442
**8.6 mM MA**	0.022581201
**21.5 mM MA**	6.7662E-07#

vs. (p-values for t-test)	Control (0 mM MA)	50 µM PD150606 (0 mM MA)
**0.86 mM MA**	8.23212E-06	0.004816851
**4.3 mM MA**	1.76517E-05	0.000188242
**8.6 mM MA**	3.62796E-07	0.000311769
**21.5 mM MA**	5.17559E-08*	0.001566528

*/#p<0.001, Student's t-test - Not every significant value has been marked with */# for better understanding of the figure.

Figure 4a:

	p-values for t-test
**Ctrl vs. Ctrl NPPB**	1.92829E-11*
**MA vs. MA NPPB**	1.32699E-05*
**Ctrl vs. MA**	1.25908E-11*
**Ctrl NPPB vs. MA NPPB**	0.068890101

*p<0.05, Student’s t-test

Figure 4b:

p-values for t-test	without vs. with Cl
**Control**	2.99577E-05*
**0,86 mM MA**	3.90014E-08*
**4,3 mM MA**	2.01798E-05*
**8,6 mM MA**	5.86787E-05*
**21,5 mM MA**	0.001530277*

*p<0.05, Student’s t-test
